# Dark-field chest radiography signal characteristics in inspiration and expiration in healthy and emphysematous subjects

**DOI:** 10.1186/s41747-025-00578-x

**Published:** 2025-03-27

**Authors:** Theresa Urban, Florian T. Gassert, Manuela Frank, Rafael Schick, Henriette Bast, Jannis Bodden, Alexander W. Marka, Lisa Steinhelfer, Manuel Steinhardt, Andreas Sauter, Alexander Fingerle, Gregor S. Zimmermann, Thomas Koehler, Marcus R. Makowski, Daniela Pfeiffer, Franz Pfeiffer

**Affiliations:** 1 Chair of Biomedical Physics, Department of Physics, School of Natural Sciences, Technical University of Munich, 85748 Garching, Germany; 2 Munich Institute of Biomedical Engineering, Technical University of Munich, 85748 Garching, Germany; 3 Department of Diagnostic and Interventional Radiology, School of Medicine & Klinikum rechts der Isar, Technical University of Munich, 81675 Munich, Germany; 4 Department of Neuroradiology, School of Medicine & Klinikum rechts der Isar, Technical University of Munich, 81675 Munich, Germany; 5 Division of Respiratory Medicine, Department of Internal Medicine I, School of Medicine & Klinikum rechts der Isar, Technical University of Munich, 81675 Munich, Germany; 6 Philips Innovative Technologies, 22335 Hamburg, Germany; 7 Munich Institute for Advanced Study, Technical University of Munich, 85748 Garching, Germany

**Keywords:** Emphysema, Lung, Radiography (thoracic), Respiration

## Abstract

**Background:**

Dark-field chest radiography is sensitive to the lung alveolar structure. We evaluated the change of dark-field signal between inspiration and expiration.

**Methods:**

From 2018 to 2020, patients who underwent chest computed tomography (CT) were prospectively enrolled, excluding those with any lung condition besides emphysema visible on CT. Participants were imaged in both inspiration and expiration with a prototype dark-field chest radiography system. We calculated the total dark-field signal ∑DF and the dark-field coefficient *ϵ*, assumed to be proportional to the total number of alveoli and the alveolar density, respectively.

**Results:**

Eighty-eight subjects, aged 64 years ± 11 (mean ± standard deviation), 55 males, were enrolled. Dark-field signal in the lung projection appeared higher in expiration compared to inspiration. Over all participants, ∑DF was higher in inspiration (1.6 × 10^-2^ ± 0.4 × 10^-2^ m^2^) compared to expiration (1.5 × 10^-2^ ± 0.4 m^2^) (*p* < 0.001), with its expiration-to-inspiration not ratio being different for any emphysema subgroup. The dark-field coefficient *ϵ* was lower in inspiration (2.3 ± 0.6 m^-1^) compared to expiration (3.1 ± 1.1 m^-1^) (*p* < 0.001) over all participants. The dark-field coefficient in inspiration and expiration, as well as their ratio, was lower for at least moderate emphysema when compared to the control group (*e.g*., *ϵ* = 2.5 ± 1.0 m^-1^ for moderate emphysema in expiration *versus* *ϵ* = 3.6 ± 0.7 m^-1^ for participants without emphysema (*p* = 0.003).

**Conclusion:**

The dark-field signal depends on the breathing state. Differences between breathing states are influenced by emphysema severity.

**Relevance statement:**

The patient’s breathing state influences the dark-field chest radiograph, potentially impacting its diagnostic value.

**Key Points:**

- Signal characteristics in dark-field chest radiography change between inspiration and expiration.
- The total dark-field signal decreases slightly from inspiration to expiration, while the dark-field coefficient increases substantially.
- The ratio of the total dark-field signal between expiration and inspiration is independent of emphysema severity, whereas the ratio of the dark-field coefficient depends on emphysema severity.

**Graphical Abstract:**

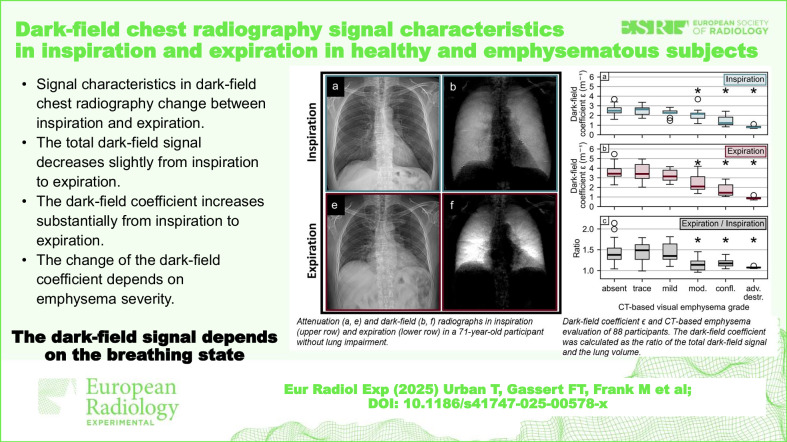

## Introduction

X-ray dark-field imaging is an emerging technique that has initially been introduced in 2008 as an experimental approach [1]. As opposed to attenuation-based conventional radiography, the contrast in x-ray dark-field radiography is generated by ultra-small-angle scattering of x-rays taking place at microstructural material interfaces within the specimen under investigation [2, 3]. Healthy lungs create a relatively high signal due to their great number of air-tissue interfaces in the alveoli. Various preclinical studies in specimens and animals have shown the potential of the method for the assessment of pulmonary diseases, including pulmonary emphysema [4, 5], pulmonary fibrosis [6], radiation-induced lung damage [7, 8], lung tumors [9], and pneumothorax [10].

The previously mentioned studies were conducted in either animals or human cadavers. Recently, dark-field chest radiography has been successfully translated into the first clinical explorations on humans [11, 12]. After the qualitative and quantitative characteristics of dark-field images had been analyzed in healthy humans [11], the potential of the technique for the assessment of various pulmonary diseases has also been shown in humans. Those studies found a reduction of dark-field signal either due to a lower number of alveolar walls—as in emphysema [12–14], combined pulmonary fibrosis and emphysema [15], and lymphangioleiomyomatosis [16]—or due to the filling of alveolar spaces with inflammatory cells and fluids—as in COVID-19 pneumonia [17].

However, all the previously mentioned studies only analyzed dark-field images in full inspiration.

A study in mechanically ventilated *in vivo* mice found changing dark-field signals with breathing cycles [18], and a study on *in vivo* pigs found a strong dependence of the dark-field signal on ventilation pressure [19]. An *ex vivo* study in humans found that the dark-field signal of the lungs increases with higher pressure levels when inflating a human cadaver, suggesting the reopening of alveoli, which had collapsed due to post-mortem changes [20]. However, so far, the dependence of the dark-field signal on the breathing state in humans has not yet been addressed. Nevertheless, the patient’s breathing state might influence the diagnostic value of the obtained dark-field radiographs. If so, it is yet to be determined which breathing state optimizes the diagnostic value.

Therefore, the purpose of this study was to explore the signal dependency of dark-field radiographs in expiration compared to inspiration images in both healthy participants and in patients with pulmonary emphysema.

## Methods

### Participants

**Fig. 1:**
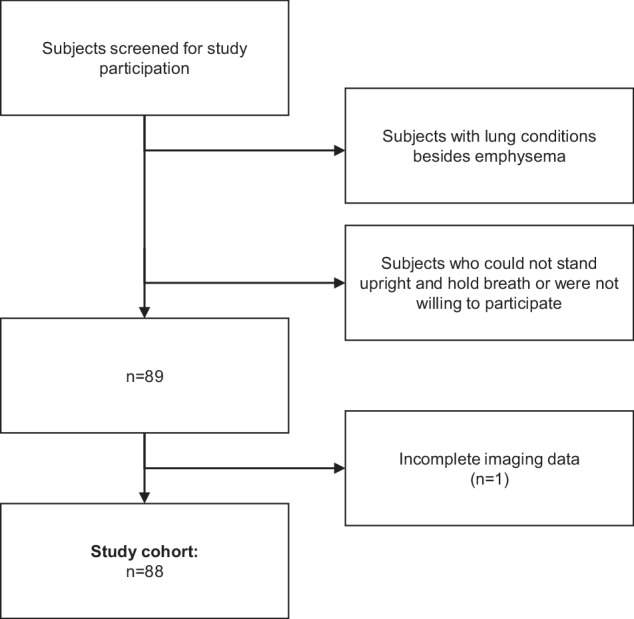
Flowchart shows participant selection criteria. Between October 2018 and October 2020, 88 participants were included in the study

This prospective study was conducted in accordance with the Declaration of Helsinki (as revised in 2013). Approval of the Institutional Review Board and National Radiation Protection Agency was obtained prior to this study (Ethics Commission of the Medical Faculty, Technical University of Munich, Germany; reference no. 166/20S). Participants gave their written informed consent. Between October 2018 and October 2020, patients of at least 18 years of age who underwent chest CT as part of their diagnostic workup were screened for study participation. Inclusion criteria were the ability to consent and the ability to stand upright without help. Exclusion criteria were any lung condition besides emphysema. Also, all participants with incomplete imaging data (*e.g*., missing lateral dark-field chest radiography) were excluded. Figure 1 shows the study flow and selection process. 39 participants and 81 participants were previously described in studies that quantitatively evaluated the characteristics of dark-field chest radiography in healthy humans [11] and for emphysema assessment [13], respectively. These previous studies investigated only dark-field images in full inspiration.

### Dark-field imaging: acquisition and analysis

All participants were examined with a prototype dark-field chest radiography system, as described previously [11–13]. It operates at 70 kVp tube voltage, with one image acquisition taking about 7 s. We performed one acquisition each in posteroanterior and lateral orientation at both full inspiration and full expiration for all participants included in this study. Participants were told to hold their breath in either inspiration or expiration with an automated prerecorded voice command directly before the acquisition.

Besides a qualitative evaluation the dark-field signal, *i.e*., the pixel values in the dark-field images, we calculated both the total dark-field signal ∑DF and the dark-field coefficient *ϵ* for each participant in both inspiration and expiration for quantitative analysis.

For the total dark-field signal ∑DF, the entire lung was segmented in the posteroanterior view and the total dark-field signal was calculated as the sum of the dark-field signal in this segmentation. This total dark-field signal is assumed to be proportional to the number of aerated alveoli in the lung. Gassert et al found a correlation of total dark-field signal and each participant’s lung volume [11].

The dark-field coefficient *ϵ* was calculated for the entire lung as the ratio of the total dark-field signal and the lung volume of the respective participant, as described previously [11, 13]. Lung volume was derived from the attenuation-based posteroanterior and lateral radiographs as described by Pierce et al [21]. The dark-field coefficient describes the average dark-field signal generated per length of lung tissue and depends on the microstructural properties of the tissue, such as the packing density of the alveoli and, therefore, the breathing state [19].

The relative lung volume difference between inspiration and expiration was calculated as the difference in lung volume between inspiration and expiration relative to the lung volume in inspiration: (*V*_insp_ − *V*_exp_)/*V*_insp_.

### CT-based emphysema evaluation

CT was performed on one of two CT scanners (iCT and IQon Spectral CT, Philips Healthcare, Amsterdam, The Netherlands) with the following parameters according to routine clinical protocols: collimation 128 × 0.625 mm or 64 × 0.625 mm for iCT and IQon, respectively; slice spacing 0.4 mm and 0.3 mm, respectively; pitch factor 0.8 and 0.9, respectively; tube voltage 120 kVp for both; modulated tube current 102–132 mA. Images were reconstructed at 0.9 mm slice thickness with a lung-specific convolution kernel.

The semiquantitative assessment of emphysema based on CT images was determined by three radiologists: J.B., A.S., A.F., with 4, 7, and 13 years of experience in CT imaging, with AF also having subspecialty experience in thoracic radiology. Participants were either classified as non-emphysematous (absent) or rated on a five-point scale grading system (trace, mild, moderate, confluent, advanced destructive emphysema) using the Fleischner Society guidelines for emphysema scoring [22]. We treated the gradings as a Likert scale and used the median of the three readers as the ground truth of the participant’s emphysema severity.

### Statistical analysis

All statistical analyses were performed using R version 3.2.4 (R Foundation for Statistical Computing, Vienna, Austria). A *p*-value of less than 0.05 was considered to indicate a statistically significant difference. The participant parameters age, weight, height, and lung volume were tested for significant differences between participants with and without emphysema with Student *t*-test. For the parameter sex, a *χ*^2^ test was used. The total dark-field signal in inspiration, in expiration, and its ratio of expiration and inspiration for the different emphysema severity groups to the group without emphysema were compared using the Wilcoxon-Mann–Whitney *U*-test. The same comparison was repeated for the dark-field coefficient in inspiration, expiration, and its ratio of expiration and inspiration. The differences in the total dark-field signal and the dark-field coefficient between inspiration and expiration were analyzed using the paired Wilcoxon test.

## Results

### Participants

We studied 88 participants (55 males, 33 females), aged 64 ± 11 years (mean ± standard deviation), with a height of 170 ± 7 cm and weight 75 ± 15 kg (Table 1). Of the 52 patients with emphysema, 21 were graded as trace, 11 as mild, 9 as moderate, 7 as confluent, and 4 as advanced destructive.

**Table 1:** Subject demographics

Parameter	All	Absent	Emphysema	*p*-value
Number of participants	88	36	52	−
Men/Women	55/33	22/14	33/19	0.823
Age (years)	64 ± 11	63 ± 11	66 ± 11	0.212
Weight (kg)	75 ± 15	80 ± 16	71 ± 14	**0.005**
Height (cm)	170 ± 7	169 ± 7	170 ± 8	0.629
Total lung volume (liters)				
Inspiration (Insp)	7.3 ± 1.7	6.8 ± 1.4	7.6 ± 1.8	**0.023**
Expiration (Exp)	5.2 ± 1.8	4.5 ± 1.0	5.7 ± 2.0	**<** **0.001**
Difference Insp-Exp	2.1 ± 1.1	2.3 ± 1.0	2.0 ± 1.2	0.163
Relative difference in lung volume	0.29 ± 0.14	0.33 ± 0.11	0.27 ± 0.16	**0.022**

Values are given as mean ± standard deviation. No emphysema = “absent”; Emphysema = “trace”, “mild”, “moderate”, “confluent”, or “advanced destructive” according to the Fleischner society grading system [22]. The relative difference in lung volume was calculated as the ratio between the difference in lung volume between inspiration and expiration and the lung volume in inspiration. The *p*-values for the significance of differences between the no emphysema group and the emphysema group are listed in the very right column. Significant *p*-values are marked in bold

### Qualitative analysis

**Fig. 2:**
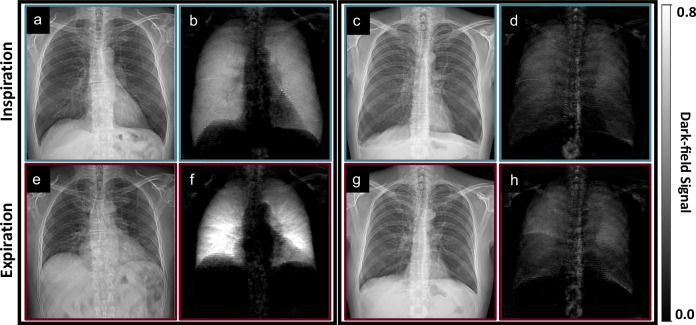
Attenuation (**a**, **c**, **e**, **g**) and dark-field (**b**, **d**, **f**, **h**) radiographs in inspiration (upper row) and expiration (lower row) in a 71-year-old healthy participant (lung volume in inspiration: 8.9 L; left array) and a 55-year old participant with advanced destructive emphysema (lung volume in inspiration: 10.6 L; right array). The emphysema patient exhibits typical signs of hyperinflation in the conventional radiograph and a lower dark-field signal in the dark-field radiograph when compared to the healthy participant. While the lung area decreases from inspiration to expiration images, the dark-field signal intensity increases. Both effects appear to be weaker for the emphysema patient. The same window and level settings were applied within the respective modality for all participants

Figure 2 shows example attenuation-based and dark-field radiographs of a 71-year old healthy participant with no emphysema (“absent”) and a 55-year-old participant with advanced destructive emphysema. The qualitative characteristics of inspiration have been described previously for healthy participants [11] and patients with pulmonary emphysema [13]. The participant with emphysema shows secondary signs of hyperinflation, including flattened hemidiaphragms, irregular parenchymal radiolucency, and a paucity of blood vessels. He also exhibits a lower dark-field signal in both inspirational and expirational dark-field images when compared to the healthy participant. In conventional radiographs, the hemidiaphragm shadows move up between inspiration and expiration, and thus, the area of the lung on the two-dimensional radiographs decreases. In dark-field radiographs, the area of the lung, characterized by a high dark-field signal compared to surrounding tissues, also decreases. While a decrease in lung area can be observed in both the healthy participant and the patient with emphysema, this effect appears to be weaker for the latter. Also, particularly with respect to the healthy participant, the dark-field signal increases between inspiration and expiration, with a predominance in the lower zones.

### Lung volume

Over all participants, the lung volume in inspiration was 7.3 ± 1.7 L (mean ± standard deviation) and decreased to 5.2 ± 1.8 L in expiration (*p* < 0.001) (Table 1). The lung volume was greater in emphysema patients compared to non-emphysema participants (‘absent’) in both inspiration (*p* = 0.023) and expiration (*p* < 0.001). The relative difference in lung volume was greater in the absent group (0.33 ± 0.11) compared to emphysema patients (0.27 ± 0.16, *p* = 0.022).

### Total dark-field signal

**Fig. 3:**
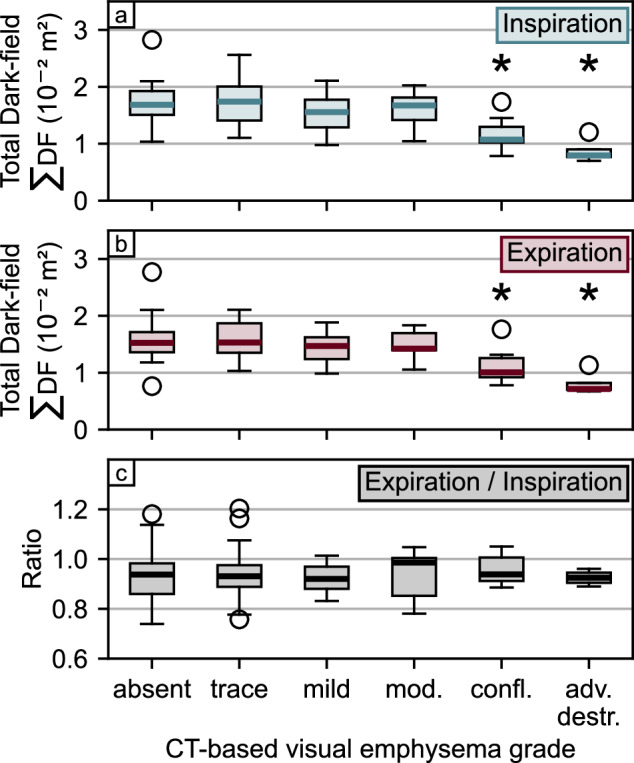
Total dark-field signal and CT-based emphysema evaluation of 88 participants. The total dark-field signal was calculated as the sum of the dark-field signal of the entire lung of each participant. Comparison of total dark-field signal in inspiration (**a**) and expiration (**b**) as well as of the ratio between expiration and inspiration (**c**) with visual emphysema grades determined from CT assessment according to the Fleischner scale. The asterisk indicates significant difference (*p* < 0.05) compared with the absent group. CT, Computed tomography

Over all participants, the total dark-field signal ∑DF was higher in inspiration (∑DF = 1.6 × 10^-2^ ± 0.4 m^2^) compared to expiration (∑DF = 1.5 × 10^-2^ ± 0.4 m^2^) (*p* < 0.001). The total dark-field signal stratified by breathing state and emphysema severity levels is shown in Fig. 3 and Table 2.

**Table 2:** Total dark-field signal ∑DF

Emphysema severity	Absent	Trace	Mild	Moderate	Confluent	Adv. destr.
Number of participants	36	21	11	9	7	4
∑DF, Insp (× 10^-2^ m^2^)	1.7 ± 0.3	1.7 ± 0.4	1.6 ± 0.4	1.6 ± 0.3	1.2 ± 0.3	0.9 ± 0.2
*p*-value		0.798	0.223	0.479	**0.001**	**<** **0.001**
∑DF, Exp (× 10^-2^ m^2^)	1.6 ± 0.4	1.6 ± 0.3	1.4 ± 0.3	1.5 ± 0.2	1.1 ± 0.3	0.8 ± 0.2
*p*-value		0.862	0.187	0.809	**0.003**	**<** **0.001**
∑DF, ratio	0.93 ± 0.10	0.93 ± 0.11	0.93 ± 0.06	0.94 ± 0.09	0.96 ± 0.06	0.93 ± 0.03
*p*-value		0.941	0.950	0.403	0.429	0.879

Values are given as mean ± standard deviation. The total dark-field signal ∑DF was calculated as the sum of the dark-field signal of the entire lung. Emphysema severities are according to the Fleischner Society grading system [22]. *p*-values are given for the significance of differences between the group with respective emphysema severity and the no emphysema group (“absent”). Significant *p*-values are marked in bold. *Adv. destr*. Advanced destructive, *Exp* Expiration, *Insp* Inspiration

Compared to the control group (absent emphysema), the average total dark-field signal in the inspiration of the trace, mild, and moderate emphysema group did not decrease significantly. However, it was significantly lower for the confluent and advanced destructive group. The same applied to the total dark-field signal in expiration. The average ratio between the total dark-field signal in expiration and inspiration was 0.93 ± 0.09 over all participants. Regarding the emphysema groups, no difference was observed between any emphysema group and the control group, and the ratio ranged between 0.93 and 0.96 (*p* ≥ 0.403 for all).

### Dark-field coefficient

**Fig. 4:**
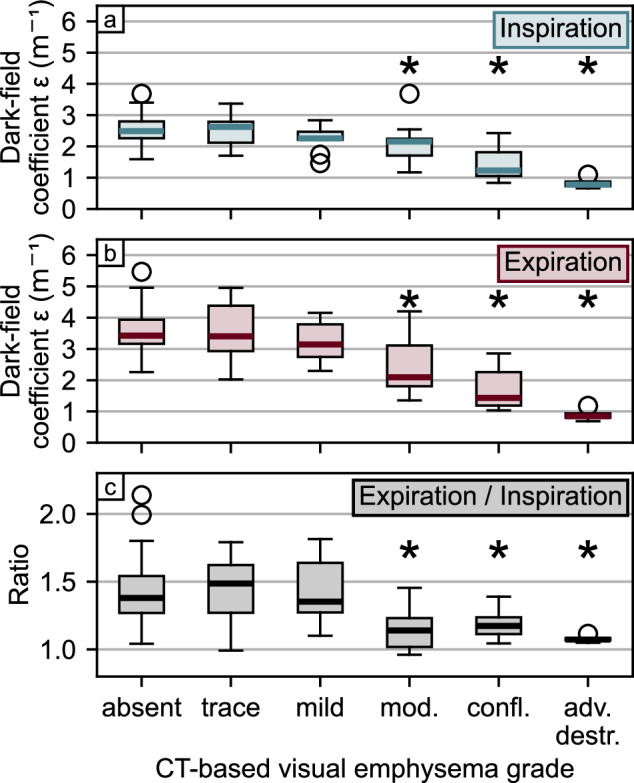
Dark-field coefficient *ϵ* and CT-based emphysema evaluation of 88 participants. The dark-field coefficient was calculated as the ratio between the total dark-field signal and the lung volume derived from conventional radiographs for each participant. Comparison of the dark-field coefficient in inspiration (**a**) and expiration (**b**) as well as of the ratio between expiration and inspiration (**c**) with visual emphysema grades determined from CT assessment according to the Fleischner scale. The asterisk indicates significant difference (*p* < 0.05) compared with the absent group. CT, Computed tomography

Over all participants, the dark-field coefficient *ϵ*, calculated as the ratio between total dark-field signal and lung volume, was higher in expiration (*ϵ* = 3.1 ± 1.1 m^-1^) compared to inspiration (*ϵ* = 2.3 ± 0.6 m^-1^) (*p* < 0.001). The dark-field coefficient stratified by breathing state and emphysema severity levels is shown in Fig. 4 and Table 3.

**Table 3:** Dark-field coefficient *ϵ*

Emphysema severity	Absent	Trace	Mild	Moderate	Confluent	Adv. destr.
Number of participants	36	21	11	9	7	4
*ϵ*, Insp (m^-1^)	2.5 ± 0.4	2.5 ± 0.4	2.3 ± 0.4	2.1 ± 0.7	1.5 ± 0.6	0.8 ± 0.2
*p*-value		0.836	0.076	**0.019**	**<** **0.001**	< **0.001**
*ϵ*, Exp (m^-1^)	3.6 ± 0.7	3.6 ± 0.9	3.2 ± 0.6	2.5 ± 1.0	1.7 ± 0.8	0.9 ± 0.2
*p*-value		0.954	0.179	**0.003**	< **0.001**	< **0.001**
*ϵ*, ratio	1.4 ± 0.2	1.4 ± 0.2	1.4 ± 0.2	1.2 ± 0.2	1.2 ± 0.1	1.1 ± 0.03
*p*-value		0.772	0.755	**0.003**	**0.008**	**0.001**

Values are given as mean ± standard deviation. The dark-field coefficient *ϵ* was calculated as the ratio between the total dark-field signal and the lung volume. Emphysema severities are according to the Fleischner Society grading system [22]. *p*-values are given for the significance of differences between the group with respective emphysema severity and the no emphysema group (“absent”). Significant *p*-values are marked in bold. *Adv. destr*. Advanced destructive, *Exp* Expiration, *Insp* Inspiration

In both breathing states, no differences were found for the emphysema groups trace and mild when compared with the control groups (absent emphysema). However, it was lower for the moderate, confluent, and advanced destructive emphysema groups in both inspiration and expiration, respectively.

The ratio between the dark-field coefficient in expiration to the dark-field coefficient in inspiration was 1.42 ± 0.24 for the control group (absent emphysema) and did not change for the trace and mild emphysema group. Compared to the control group (absent emphysema), the ratio was lower for the moderate, confluent, and advanced destructive emphysema groups.

## Discussion

In this work, we investigated the signal characteristics of the dark-field signal in both inspiration and expiration in 88 participants with different grades of emphysema severity. In a qualitative image evaluation, we found a higher dark-field signal in expiration, and we found that this signal increase was stronger in patients without emphysema. Furthermore, we quantitively evaluated both the total dark-field signal ∑DF and the dark-field coefficient *ϵ*. The dark-field coefficient *ϵ* increased by 40% to 43% from inspiration to expiration in patients without, with trace, or with mild emphysema. In patients with more severe emphysema stages, the dark-field coefficient *ϵ* increased only by 8% to 18%. In contrast, the average ratio between the total dark-field signal ∑DF in expiration and inspiration was 0.93 ± 0.09 and did not differ significantly between the absent and emphysema groups.

The findings in this work are consistent with previous studies on the effect of breathing state or ventilation pressure on dark-field radiographs, which were performed in mechanically ventilated mice [18] and pigs [19]. These studies also found an increase in dark-field signals with lower ventilation pressures and a predominance of the increase in the lower lung. This predominance suggests that the alveolar packing density increases stronger in the lower lung areas, which is in line with the fact that lower lungs physiologically show greater volume changes between inspiration and expiration than upper lungs because of the influence of gravity on lung recoil and apicobasal movement of the adjacent diaphragm during inspiration [23].

For all participants, the total dark-field signal in expiration was lower compared to inspiration, with a ratio of 0.93 ± 0.09. We would expect the total dark-field signal to remain the same, as the number of alveoli remains constant [24]. Therefore, the finding that the ratio slightly differs from 1 needs further investigation.

The decrease of the dark-field coefficient *ϵ* with emphysema severity in inspiration has been reported before [13] in a smaller patient group that was also included in this work. We now found that this decrease in *ϵ* with increasing emphysema severity is also present when analyzing images in expiration. We further found a general increase of *ϵ* with expiration when compared to inspiration, regardless of emphysema severity. This finding is consistent with the dependence of *ϵ* on the alveolar density, as the packing density of the alveoli increases in expiration. In other words: as *ϵ* is calculated as the ratio of the total dark-field signal ∑DF and the lung volume, lower lung volumes (as present in expiration) lead to a higher *ϵ* when assuming a constant total dark-field signal ∑DF. However, the increase in *ϵ* from inspiration to expiration was much higher in the groups without, with trace, or with mild emphysema (about 40% increase) than in the groups with more severe emphysema stages (8% to 18% increase). The reason for this is probably that patients with more severe emphysema have a limited capability to breathe out due to expiratory flow limitation, the pathophysiological hallmark of COPD [25].

With regard to the quantitative detection of emphysema using the dark-field coefficient, the performance of inspiration and expiration images was similar. The dark-field coefficient in both breathing states showed a significant difference between the absent emphysema group and the groups with at least moderate emphysema. To answer the question of which breathing state is favorable for dark-field radiography, it will be necessary to conduct qualitative analyses with reader studies in addition to the quantitative evaluations presented here. Also, more studies on different lung pathologies will be needed before a recommendation can be given.

As the dark-field coefficient *ϵ* is not only sensitive to the total dark-field signal, which is determined by the number of alveoli (and thus depending on the emphysema stage) but also to the lung volume (and thus depending on the breathing state), special care has to be given to accurate breathing instructions for the patient. So far, it doesn’t seem necessary to monitor the participant’s breathing state with, *e.g*., *in situ* pulmonary function tests during image acquisition, as there are significant differences between the dark-field coefficients of groups with different emphysema stages without doing so. Nevertheless, studies with *in situ* pulmonary function tests could yield even higher effect sizes and significant differences at earlier emphysema stages.

This study has limitations. First, even though patients were told to hold their breath in full inspiration or expiration, the breathing state was not measured quantitatively. Therefore, we can not be sure if all patients were really imaged in full inspiration or expiration, especially since patients in more severe emphysema stages might have had difficulties holding their breath for 7 s in full expiration. If some participants were indeed not imaged in full inspiration or expiration, we would underestimate the actual changes in the dark-field coefficient between breathing states. Second, even though pulmonary function tests are the standard for lung volume determination, the lung volume used for the calculation of dark-field coefficients was determined from chest radiographs. During image acquisition, *in situ* pulmonary function tests were not available, and pulmonary function tests at a different point in time would potentially yield different lung volumes than those during image acquisition. Another limitation is the performance of this study at a single institution and the small number of study participants, especially in the more severe emphysema stages. Any statistical significance found in this work will have to be confirmed in further studies with larger cohorts, preferably also at other institutions. Furthermore, we investigated only healthy lungs and emphysema as lung pathology. Other diseases might have different effects on the variation of dark-field signals with breathing state.

In conclusion, we found that the dark-field signal generally depends on the breathing state. As expected—based on the assumption that the dark-field signal essentially is a measure of the number of alveoli in the beam path—the total dark-field signal over the whole lung changed only slightly (we observed a decrease by only less than 10%) between inspiration and expiration. In contrast, the dark-field coefficient, which relates more to the density of alveoli, increased by up to 40% from inspiration to expiration, especially in participants with no to mild emphysema. More studies will be necessary to enable a recommendation on the optimum breathing state for dark-field chest radiography.

## Data Availability

The datasets used and/or analyzed during the current study are available from the corresponding author upon reasonable request.

## Abbreviations

∑DF: Total dark-field signal
*ϵ*: Dark-field coefficient
